# Assessment of multi-parameter dual-energy CT in predicting muscle invasion in bladder cancer: comparison with VI-RADS

**DOI:** 10.1186/s13244-026-02275-8

**Published:** 2026-04-08

**Authors:** Mingyang Sun, Jian Wang, Jiawen Luo, Shengyuan Lai, Lei Wang, Yajie Liu, Xueling Wen, Caiyun Yu

**Affiliations:** Department of Radiology, The Second Hospital of Dalian Medical University, Dalian, People’s Republic of China

**Keywords:** Dual-energy Computed Tomography, Muscle-invasive bladder cancer, Vesical Imaging-Reporting and Data System

## Abstract

**Objectives:**

Preoperative differentiation of muscle-invasive bladder cancer (MIBC) is challenging. This study explores the application of the Dual-Energy CT (DECT) model and the vesical imaging reporting and data system (VI-RADS) in assessing MIBC and compares their diagnostic performance.

**Materials and methods:**

This single-center prospective study included 105 patients (33 MIBC and 72 non-MIBC cases). Two radiologists independently performed DECT morphological assessment, and evaluated quantitative DECT parameters and VI-RADS scores, blinded to the pathological findings. A DECT-based model was constructed by integrating independent quantitative predictors with tumor diameter (*D*). Diagnostic performance was compared using the area under the receiver operating characteristic curve (AUC). Subgroup analyses were performed for equivocal VI-RADS 3–4 cases and small lesions (*D* < 3 cm).

**Results:**

VI-RADS achieved an AUC of 0.926 (sensitivity 0.970) but low specificity (0.542). The DECT model showed moderate performance (AUC = 0.761). However, the DECT + D model achieved an AUC of 0.925, comparable to VI-RADS (*p* > 0.05), with improved specificity (0.833). Crucially, in equivocal VI-RADS 3–4 cases, DECT + D (AUC = 0.904) significantly outperformed VI-RADS (AUC = 0.652, *p* < 0.05), while standalone DECT achieved an AUC of 0.824. For *D* < 3 cm, DECT + D (AUC = 0.895) and DECT (AUC = 0.857) were comparable to VI-RADS (AUC = 0.866), but DECT maintained high specificity (0.934). A nomogram and web-based risk calculator were developed.

**Conclusion:**

The DECT-based model achieves diagnostic accuracy comparable to VI-RADS and demonstrates superior stability in clinically challenging subgroups. It serves as a robust, objective alternative for preoperative bladder cancer staging, particularly beneficial for patients with MRI contraindications.

**Critical relevance statement:**

This study demonstrates that a quantitative DECT-based model provides an accurate, objective alternative to MRI for preoperative bladder cancer staging, mitigating diagnostic ambiguity in clinically equivocal cases and ensuring accurate risk stratification in small lesions.

**Key Points:**

- Preoperative differentiation of muscle invasion is critical for bladder cancer management.
- The DECT-based model yields diagnostic accuracy statistically comparable to VI-RADS.
- The DECT-based model improves diagnostic confidence in VI-RADS 3–4 lesions.

**Graphical Abstract:**

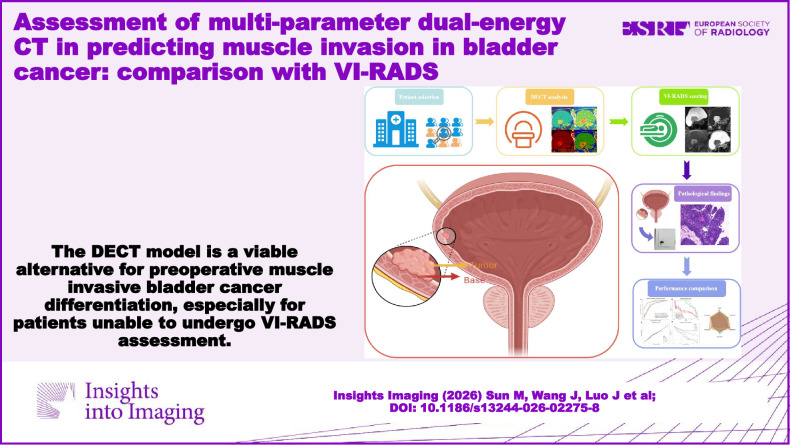

## Introduction

Bladder cancer ranks as the tenth most common malignancy worldwide and the second most prevalent tumor of the genitourinary system [1]. At initial diagnosis, approximately 75% of patients present with non–muscle-invasive bladder cancer (NMIBC), while the remaining 25% have muscle-invasive bladder cancer (MIBC) [2]. Compared with NMIBC, MIBC represents a more advanced disease stage, is associated with a higher recurrence rate, and carries a poorer prognosis [3, 4]. The standard initial treatment for NMIBC is transurethral resection of bladder tumor (TURBT), whereas MIBC typically requires radical cystectomy combined with neoadjuvant chemotherapy [5, 6]. Accurate preoperative assessment of muscle invasion is crucial for optimizing risk-stratified treatment strategies [7].

TURBT serves as the cornerstone of bladder cancer diagnosis, staging, and management by providing histopathological specimens for analysis [3, 6]. However, as an invasive procedure, TURBT is associated with complications such as catheter-related bladder irritation, hemorrhage, infection, and, in rare cases, bladder perforation [8]. Furthermore, the quality of TURBT is highly dependent on the surgeon’s expertise, and the presence or absence of detrusor muscle in the specimen is also directly related to the risk of early recurrence [9, 10]. A systematic review demonstrated a 51% risk of persistence and an 8% risk of understaging for T1 tumors [11]. This can lead to an underestimation of MIBC, delaying optimal treatment and increasing the risk of disease progression and postoperative recurrence. Therefore, there is a critical need for a noninvasive and cost-effective imaging modality to accurately assess muscle invasion in bladder cancer before surgery.

The Vesical Imaging-Reporting and Data System (VI-RADS), a standardized five-point scoring system, has been developed to improve the accuracy of bladder cancer staging, particularly in evaluating muscle invasion [12]. Multiple studies have validated the good diagnostic performance and interobserver agreement of VI-RADS scoring in detecting MIBC [13–15]. Accurate preoperative staging can be particularly challenging in patients with contraindications to MRI, such as the presence of metallic implants, severe claustrophobia, or an inability to maintain a stable position for extended periods. Dual-energy CT (DECT) employs X-ray beams at different energy levels, allowing improved differentiation of tissues or materials that may exhibit similar attenuation on conventional single-energy CT [16, 17]. The expanding clinical applications of DECT are of growing interest [18]. Previous studies have demonstrated that quantitative DECT parameters can predict lymph node metastasis in gastric cancer, microvascular invasion in hepatocellular carcinoma, and chemotherapy response in nasopharyngeal carcinoma [19–21]. However, to date, no studies have investigated the role of DECT in predicting muscle invasion in bladder cancer. We hypothesize that the DECT-based model can achieve diagnostic performance comparable to VI-RADS, providing a viable alternative for bladder cancer patients who are unable to undergo MRI.

The purpose of this study was to prospectively develop a multiparametric DECT-based model for predicting muscle invasion in bladder cancer and conduct a comparative analysis with VI-RADS.

## Materials and methods

### Patient selection

**Fig. 1:**
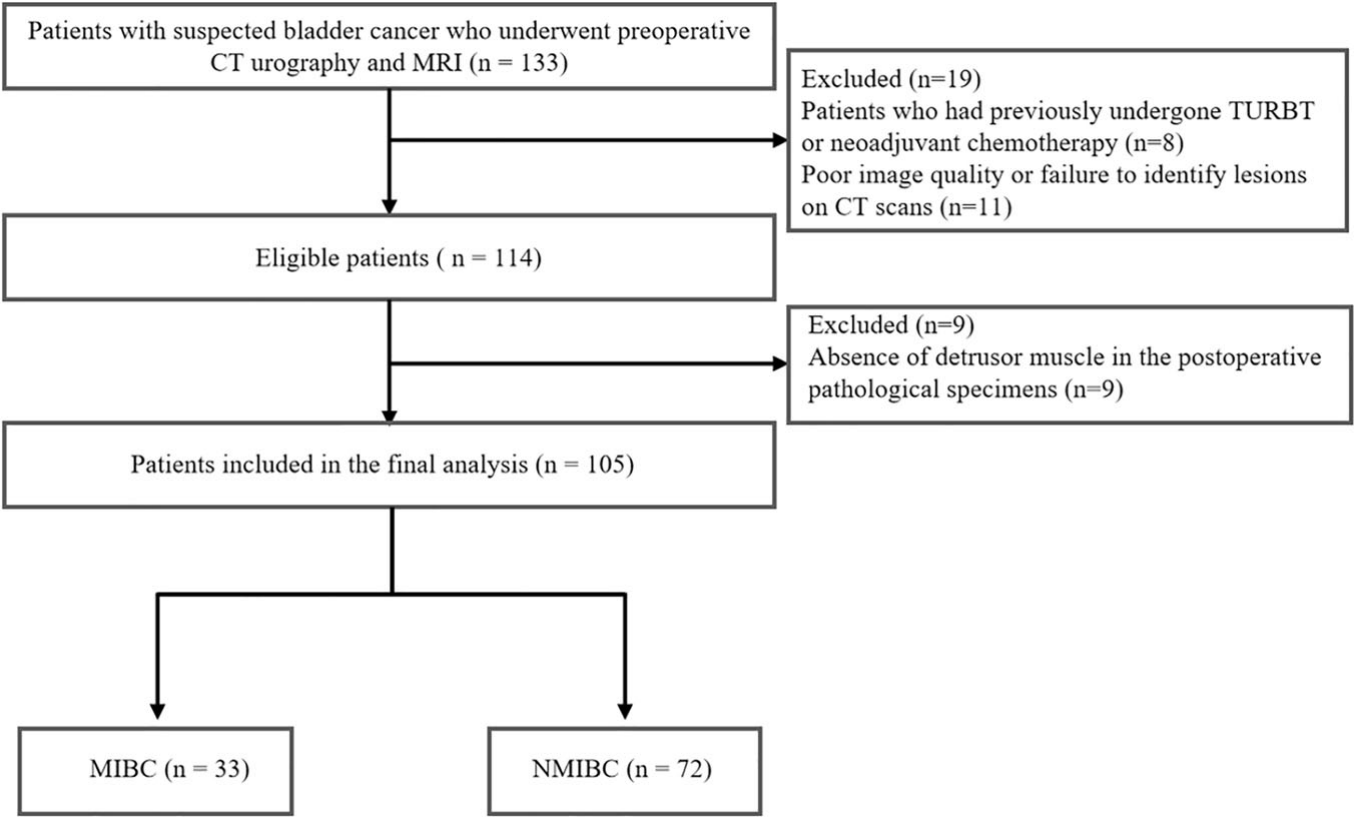
Patient selection flowchart. TURBT, transurethral resection of bladder tumor; MIBC, muscle-invasive bladder cancer; NMIBC, non-muscle-invasive bladder cancer

This prospective study was approved by the institutional ethics committee of The Second Hospital of Dalian Medical University (Approval No. KY2024-103-01-01), with a waiver of informed consent for the participants. We prospectively and consecutively enrolled patients with suspected bladder cancer who underwent preoperative CT urography (CTU) and contrast-enhanced MRI at the hospital between August 2023 and August 2024. The inclusion criteria were: (a) patients who underwent preoperative DECT and contrast-enhanced MRI; (b) histopathological confirmation of urothelial carcinoma following TURBT or cystectomy. The exclusion criteria included: (a) poor image quality or failure to identify lesions on CT scans (*n* = 11); (b) absence of detrusor muscle in the postoperative pathological specimens, precluding the assessment of muscle invasion (*n* = 9); (c) patients who had previously undergone TURBT or neoadjuvant chemotherapy as part of their follow-up (*n* = 8). Clinical data were retrieved from the picture archiving and communication system (PACS). A total of 133 patients were initially included, and after screening, 28 patients were excluded, leaving 105 patients for the final analysis. A patient selection flowchart of this study is shown in Fig. 1.

### Image acquisition

Based on the VI-RADS guideline [12], the aim of this preparation was to ensure adequate bladder distension for correct visualization of the wall and identification of the muscularis propria. For this purpose, all patients were instructed to empty their bladder 1–2 h before the examination or to drink 500–1000 mL of water 30 min prior to the scan. The target bladder filling volume was moderate to avoid overdistension, which could cause discomfort or imaging artifacts. For patients with an underfilled bladder, the scan should be repeated within 30–60 min after the patient drinks more fluid.

All CTU examinations were performed using a 256-slice spiral CT scanner (Revolution CT, GE HealthCare). Acquisition parameters included gemstone spectral imaging (GSI) mode (80/140-kVp switching), automatic exposure control (GSI Assist; noise index, 10), 80-mm collimation, pitch 0.992:1, and 0.6 s tube rotation time. Iodinated contrast medium (iohexol, 320 mg I/mL) was administered via the antecubital vein at 4 mL/s. The dose was weight-adjusted (500 mg I/kg), maximum 100 mL. Corticomedullary phase (CMP) and nephrographic phase (NP) scans were performed at 30 s and 70 s post-injection, respectively. Following these scans, patients were instructed to void, after which a diuretic was administered. Excretory phase imaging was conducted 10 min later.

**Fig. 2:**
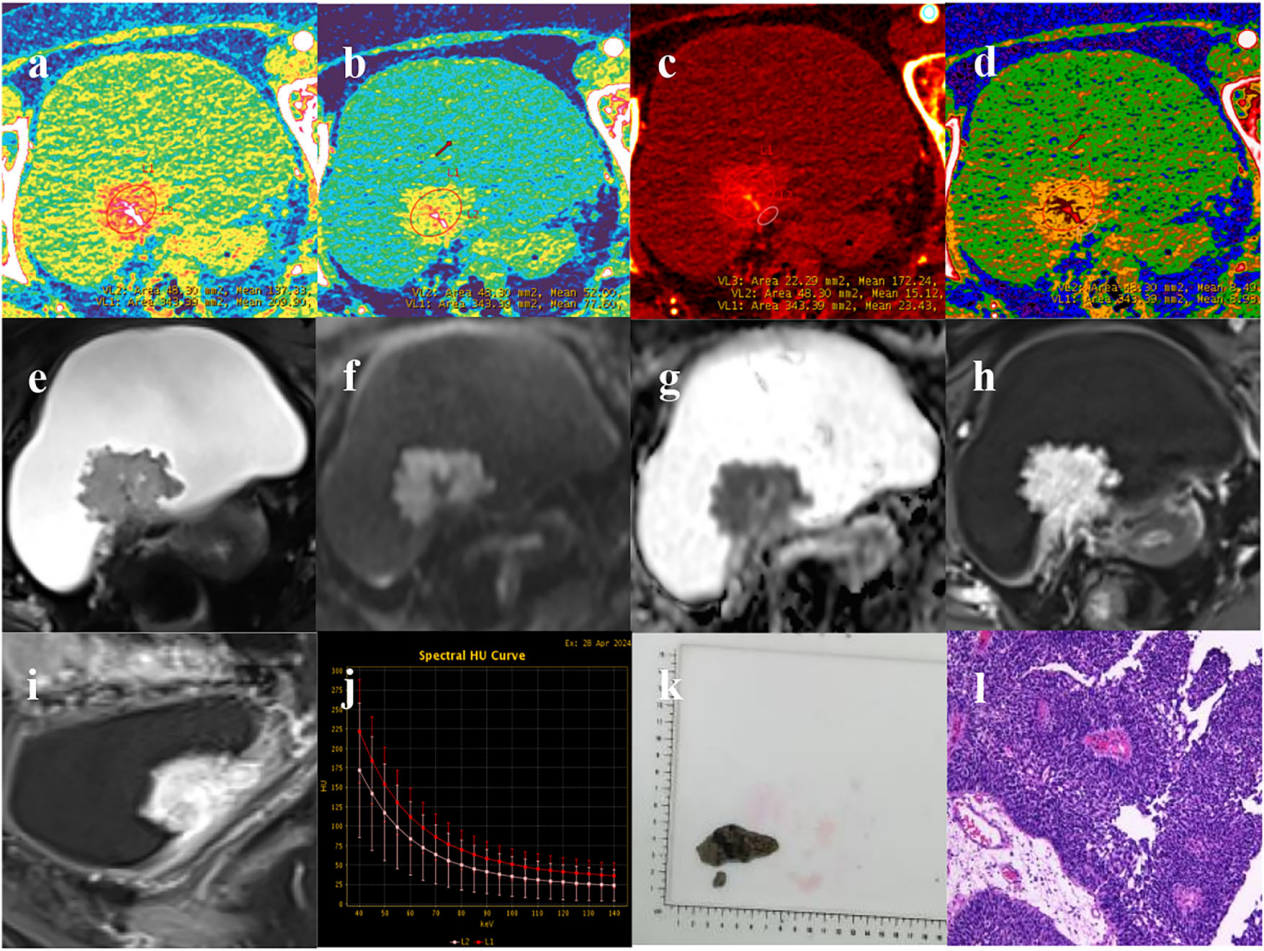
A 79-year-old female patient with pathologically confirmed NMIBC (VI-RADS 2) presenting with hematuria. Monoenergetic images (**a**) at 70 keV and (**b**) at 40 keV reveal a “cauliflower-like” mass originating from the posterior wall of the bladder. The iodine concentration map (**c**) demonstrates relatively high iodine uptake by the tumor, and the effective atomic number map (**d**) is displayed as a pseudocolor map, depicting the tumor and the stalk. On Axial T_2_-weighted imaging (T_2_WI) (**e**), the posterior bladder wall shows a slightly T2-hypointense mass with intact muscle layer signal continuity. The DWI (**f**) and ADC map (**g**) reveal the classic “Inchworm sign”, where the tumor shows restricted diffusion appearing as high signal intensity on DWI. Finally, axial DCE imaging (**h**) and sagittal DCE imaging (**i**) demonstrate marked and uniform enhancement of the tumor. Spectral curve analysis (**j**) displays two energy spectra, with the red curve representing the main tumor body and the pink curve corresponding to the tumor base. Gross specimen of the resected tumor (**k**). Histopathological examination (H&E stain, original magnification ×100) reveals high-grade papillary urothelial carcinoma with focal invasion into the subepithelial connective tissue, and no carcinoma is observed at the base (**l**)

**Fig. 3:**
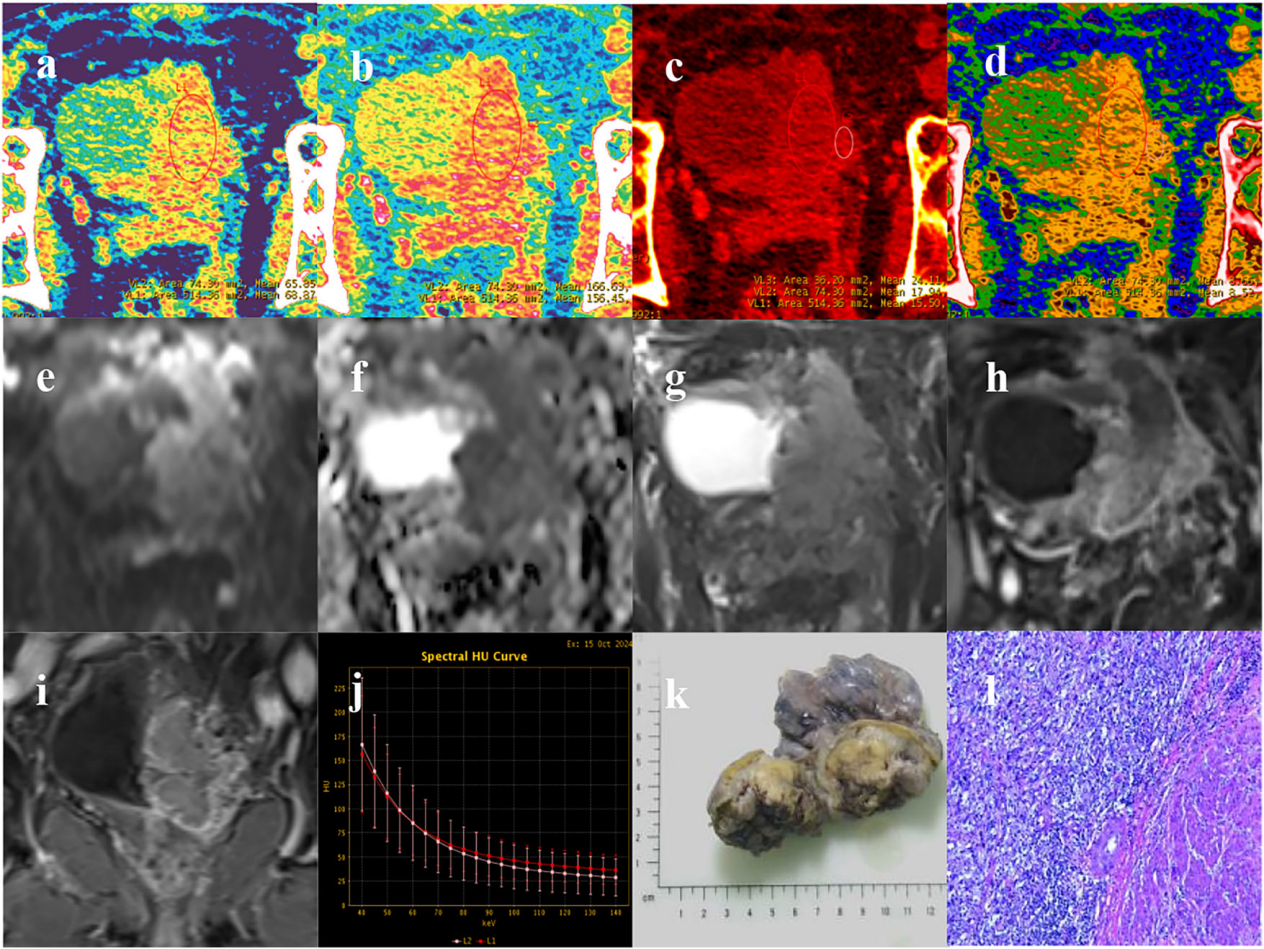
A 73-year-old male patient with pathologically confirmed MIBC (VI-RADS 5) and bilateral hydronephrosis. DECT performed at 70 keV (**a**) and 40 keV (**b**) clearly delineates a broad-based mass originating from the anterior, left, and posterior bladder walls, invading through the muscularis propria. The iodine concentration map (**c**) demonstrates markedly high iodine uptake within the tumor mass. The effective atomic number map (**d**) is displayed as a pseudocolor image, depicting a large, yellow-coded tumor mass that obstructs the left ureteral orifice and exhibits an unclear interface with the prostate, suggesting T4a invasion. Further functional assessment via DWI (**e**) shows high signal intensity, consistent with restricted diffusion observed on the ADC map (**f**). Axial T_2_WI (**g**) identifies the irregular mass as non-uniformly T2-hypointense, confirming invasion into the muscular layer and extension into the perivesical fat space. Axial DCE Imaging (**h**) demonstrates heterogeneous enhancement throughout the tumor volume. Coronal DCE imaging (**i**) clearly depicts tumor invasion into the prostate. Spectral curve analysis (**j**) compares the energy spectrum of the main tumor body (red line) against that of the tumor base (pink line). Gross specimen of the resected tumor (**k**). Histopathological examination (H&E stain, original magnification ×100) reveals high-grade invasive urothelial carcinoma demonstrating invasion into the muscularis propria (**l**)

All MRI examinations were performed using a 3.0-T magnetic resonance scanner (SIGNA Architect 3.0 T, GE HealthCare) following the institutional MRI scan parameters (Table S1). Figures 2 and 3 show DECT and MRI images of two patients.

### Quantitative DECT parameter measurements

The images were imported into the post-processing workstation (AW 4.7, GE HealthCare) and independently analyzed by Radiologist 1 and Radiologist 2, each with over five years of experience in abdominal imaging. For each phase, the CMP and NP images showing marked tumor enhancement were selected. Circular regions of interest (ROIs) were placed on both the tumor and base, defined as the approximately 5 mm-thick area where the tumor contacts the bladder wall. The same imaging slices were used across all phases within each case, avoiding regions of necrosis, hemorrhage, cystic changes, or calcification. ROI sizes were adjusted by tumor size; the largest or most advanced lesion was chosen in multifocal cases. Measurements were compared for inter-observer agreement, and the average was taken as the final value to minimize errors. Quantitative parameters were extracted from both the CMP and NP. For both the tumor and the tumor base, the following metrics were calculated : (Ⅰ) CT attenuation values of the tumor and base at 40 keV and 70 keV; (Ⅱ) spectral HU curve slope (λ_HU_); (Ⅲ) iodine concentration (IC) and normalized iodine concentration (NIC); and (Ⅳ) effective atomic number (Z_eff_). Tumor diameter (D) was defined as the longest diameter on DECT images. Nomenclature: Quantitative parameters are denoted using the format [Region]-[Phase]-[Metric]. A detailed list of all measured parameters and their abbreviations is provided in Table S2.

### VI-RADS scoring

Preoperative contrast-enhanced MRI images of each bladder cancer patient were collected. According to the VI-RADS assessment criteria, Radiologist 1 and Radiologist 2 independently performed the VI-RADS evaluation without knowledge of the pathological results. The inter-observer agreement of their scores was calculated. In cases of disagreement, a consensus was reached to determine the final result.

### DECT morphological assessment of muscle invasion

Blinded to the pathological results, Radiologist 1 and Radiologist 2 performed a qualitative evaluation of muscle invasion using DECT independently. This assessment relied on key visual criteria, including the tumor’s morphology, enhancement patterns, muscularis propria integrity, and the relationship with adjacent tissues.

Evaluation focused on growth pattern: exophytic growth with a narrow stalk suggested NMIBC; flat, broad-based, or infiltrative patterns strongly indicated MIBC. Muscularis propria integrity was subsequently assessed; a smooth, continuous layer favored NMIBC, while a discontinuous or absent layer beneath the tumor indicated contact with or penetration into the muscle layer. Mucosal enhancement line disappearance also contributed to the muscle invasion diagnosis. Furthermore, low-energy monoenergetic images enhanced tumor-structure contrast. For extravesical spread, spiculations, stranding, or irregular soft-tissue extension into perivesical fat indicated T3 disease, and direct invasion into adjacent organs signified T4 disease.

### Statistical analysis

Statistical analyses were performed using IBM SPSS Statistics (version 27.0; IBM Corporation) and R (version 4.4.2; R Foundation for Statistical Computing). Additionally, model interpretability was evaluated using SHAP analysis implemented in Python (version 3.12) with the shap package (version 0.46.0). Continuous variables were compared between two groups using the independent-samples *t*-test or Mann–Whitney *U*-test, as appropriate. Categorical variables were compared using the Chi-square test or Fisher’s exact test. The intraclass correlation coefficient (ICC) was calculated to assess the inter-observer reliability of DECT multiparametric measurements between the two radiologists. Inter-observer agreement for VI-RADS scores was assessed using quadratic-weighted Kappa (${\kappa }_{w}$). Logistic regression analysis was used to develop the DECT, D, VI-RADS, DECT + D, and VI-RADS + D predictive models. Receiver operating characteristic (ROC) curves were generated, and the area under the curve (AUC) was calculated. Differences in AUC among models were compared using the DeLong test. A formal non-inferiority analysis was conducted to compare the AUC of the DECT-based model and VI-RADS, with a pre-specified margin of −0.10. Post-hoc power analysis evaluated the sample size adequacy for this hypothesis. Model calibration was evaluated using calibration plots. Precision-recall (PR) curves were generated to assess performance under class imbalance. Clinical utility was assessed by decision curve analysis (DCA). Accuracy, precision, sensitivity, specificity, and F1 score were calculated for all predictive models and the two radiologists’ qualitative evaluations. For the predictive models, the optimal probability cutoff was determined using Youden’s *J* statistic to achieve a balanced trade-off between sensitivity and specificity. For the VI-RADS model, a score ≥ 3 was used as the fixed cutoff for MIBC, consistent with clinical practice and previous literature. Net reclassification improvement (NRI) and integrated discrimination improvement (IDI) were used to compare predictive performance between models. All 95% confidence intervals (CIs) reported in this study were calculated using 1000 bootstrap resamplings. All statistical tests were two-sided, and *p* < 0.05 was considered statistically significant.

## Results

### Clinical characteristics and VI-RADS scores of the patients

A total of 105 patients were included in this study, comprising 33 patients with MIBC (31.4%) and 72 patients with NMIBC (68.6%). Table 1 summarizes the clinical characteristics of the MIBC and NMIBC groups, with statistically significant differences (*p* < 0.05) observed between the two groups in terms of gender, VI-RADS scores, pathological grade, and D.

**Table 1:** Clinical characteristics and VI-RADS scores of patients with MIBC and NMIBC

Variables	MIBC (*n* = 33)	NMIBC (*n* = 72)	Total (*n* = 105)	*p*
Age (years)				0.103
Mean ± SD	71.2 ± 13.0	67.0 ± 10.7	68.3 ± 11.6	
Gender				0.035
Male	20 (60.6%)	59 (81.9%)	79 (75.2%)	
Female	13 (39.4%)	13 (18.1%)	26 (24.8%)	
VI-RADS scores				< 0.001
1	0 (0.0%)	5 (6.9%)	5 (4.8%)	
2	1 (3.0%)	34 (47.2%)	35 (33.3%)	
3	4 (12.1%)	22 (30.6%)	26 (24.8%)	
4	7 (21.2%)	11 (15.3%)	18 (17.1%)	
5	21 (63.6%)	0 (0.0%)	21 (20.0%)	
Number of tumors				0.476
Solitary	17 (51.5%)	44 (61.1%)	61 (58.1%)	
Multiple	16 (48.5%)	28 (38.9%)	44 (41.9%)	
Pathological grade				< 0.001
Low grade	0 (0.0%)	36 (50.0%)	36 (34.3%)	
High grade	33 (100.0%)	36 (50.0%)	69 (65.7%)	
D (cm)	3.8 ± 1.3	2.0 ± 0.9	2.5 ± 1.3	< 0.001

*MIBC* muscle-invasive bladder cancer, *NMIBC* non-muscle-invasive bladder cancer, *VI-RADS* vesical imaging reporting and data system, *D* diameter

### Consistency between DECT parameters and VI-RADS scoring

The ICCs for the DECT parameters ranged from 0.677 (95% CI: 0.563–0.786) to 0.755 (95% CI: 0.628–0.837), indicating moderate reliability (Table S3). The inter-observer agreement for VI-RADS scores was almost perfect, with a ${\kappa }_{w}$ of 0.948 (95% CI: 0.902–0.983; *p* < 0.001).

### Independent predictor selection and model building

Table 2 shows that various DECT parameters were first screened through univariable logistic regression. Among these parameters, Base-CMP-IC, Base-CMP-NIC, Tumor-CMP-λ_HU_, Base-NP-CT_40keV_, Base-NP-CT_70keV_, Base-NP-λ_HU_, Base-NP-Z_eff_, and Base-NP-IC were found to be statistically significant in the univariable analysis (*p* < 0.05). These variables were subsequently included in a multivariable logistic regression analysis, which identified Base-CMP-IC, Base-CMP-NIC, Base-NP-λ_HU_, Base-NP-Z_eff_, and Base-NP-IC as independent predictors of MIBC (*p* < 0.05). Based on these independent predictors, a DECT model was constructed. The diagnostic performance of the VI-RADS model was quantified through the incorporation of the VI-RADS score into a univariable logistic regression model and served as a comparative baseline for the DECT model.

**Table 2:** Univariable and multivariable analysis of DECT parameters for predicting MIBC

DECT parameters	MIBC (*n* = 33)	NMIBC (*n* = 72)	Univariable	Multivariable
Tumor-CMP-CT_40keV_ (HU)	155.9 ± 47.6	161.2 ± 54.5	0.627	
Tumor-CMP-CT_70keV_ (HU)	68.1 ± 15.8	70.0 ± 18.6	0.608	
Tumor-CMP-λ_HU_	-2.9 ± 1.1	-3.0 ± 1.3	0.651	
Tumor-CMP-Z_eff_	8.4 ± 0.4	8.5 ± 0.4	0.388	
Tumor-CMP-IC (mg/mL)	1.6 ± 0.6	1.6 ± 0.7	0.540	
Tumor-CMP-NIC (%)	12.6 ± 4.5	13.3 ± 5.5	0.545	
Base-CMP-CT_40keV_ (HU)	134.1 ± 46.5	115.1 ± 52.5	0.081	
Base-CMP-CT_70keV_ (HU)	57.2 ± 16.9	50.2 ± 19.6	0.083	
Base-CMP-λHU	-2.6 ± 1.1	-2.2 ± 1.2	0.103	
Base-CMP-Z_eff_	8.4 ± 0.4	8.2 ± 0.2	0.106	
Base-CMP-IC (mg/mL)	1.5 ± 0.5	1.1 ± 0.6	0.011^*^	0.006^*^
Base-CMP-NIC (%)	12.1 ± 4.2	9.1 ± 5.4	0.010^*^	0.016^*^
Tumor-NP-CT_40keV_ (HU)	210.7 ± 71.8	183.2 ± 59.0	0.050	
Tumor-NP-CT_70keV_ (HU)	85.7 ± 25.3	77.2 ± 21.2	0.084	
Tumor-NP-λHU	-4.2 ± 1.6	-3.5 ± 1.3	0.045^*^	0.156
Tumor-NP-Z_eff_	8.8 ± 0.4	8.6 ± 0.3	0.071	
Tumor-NP-IC (mg/mL)	2.2 ± 0.9	1.9 ± 0.7	0.070	
Tumor-NP-NIC (%)	52.6 ± 23.2	55.2 ± 18.6	0.529	
Base-NP-CT_40keV_ (HU)	190.7 ± 77.1	135.9 ± 66.4	0.002^*^	0.096
Base-NP-CT_70keV_ (HU)	76.2 ± 29.1	56.5 ± 24.6	0.002^*^	0.120
Base-NP-λ_HU_	-3.8 ± 1.6	-2.6 ± 1.5	0.003^*^	0.009^*^
Base-NP-Z_eff_	8.7 ± 0.4	8.4 ± 0.4	0.002^*^	0.030^*^
Base-NP-IC (mg/mL)	2.1 ± 0.9	1.4 ± 0.7	< 0.001^*^	0.016^*^
Base-NP-NIC (%)	48.6 ± 22.6	41.4 ± 18.6	0.092	
*D* (cm)	3.8 ± 1.3	2.0 ± 0.9	< 0.001^*^	< 0.001^*^

The nomenclature follows the format [Region]–[Phase]–[Metric]

### Performance comparison of the models

**Fig. 4:**
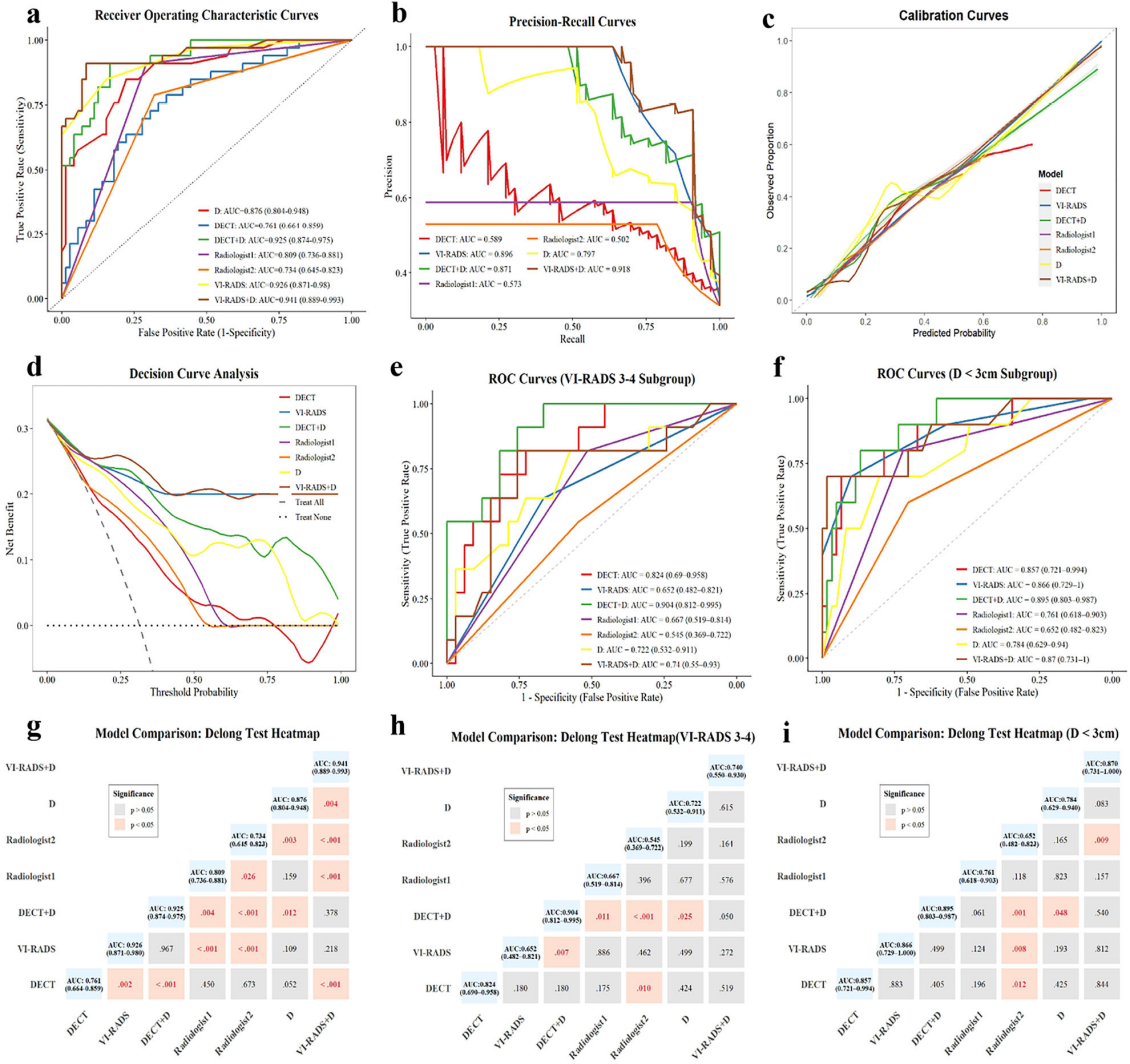
Comprehensive performance analyses for bladder cancer muscle invasion assessment: (**a**) ROC curves, (**b**) PR curves, (**c**) calibration curves, (**d**) DCA, (**e**) VI-RADS 3–4 subgroup ROC curves, (**f**) *D* < 3 cm subgroup ROC curves, (**g**) Delong test heatmap, (**h**) VI-RADS 3–4 subgroup Delong test heatmap, and (**i**) *D* < 3 cm subgroup Delong test heatmap

Figure 4a summarizes the AUC values for models and radiologists. The highest numerical AUC was observed for the VI-RADS + D model (0.941; 95% CI: 0.889–0.993), followed by the VI-RADS model (0.926; 95% CI: 0.871–0.980) and the DECT + D model (0.925; 95% CI: 0.874–0.975). The DECT model exhibited modest discriminative ability (AUC = 0.761; 95% CI: 0.664–0.859), performing comparably to Radiologist 1 (0.809; 95% CI: 0.736–0.881) and Radiologist 2 (0.734; 95% CI: 0.645–0.823).

The DeLong test heatmap (Fig. 4g) demonstrated comparative differences among the models. The DECT model demonstrated modest discriminative capacity, significantly inferior to the high-performing combined models and the standalone VI-RADS model (*p* < 0.05). However, the DECT model’s AUC was statistically comparable to those of both radiologists’ assessments (Radiologist 1, *p* > 0.05; Radiologist 2, *p* > 0.05). The single D parameter (AUC 0.876) was significantly different from the DECT + D model (AUC 0.925; *p* < 0.05), demonstrating the incremental value of DECT parameters. This incremental value was further corroborated by significant IDI (0.113; *p* < 0.05) and NRI (0.982; *p* < 0.05) (Figs. S1 and S2). Crucially, integrating D significantly improved the DECT model’s performance, elevating the DECT + D AUC to 0.925. This combined model achieved predictive power statistically indistinguishable from the optimal VI-RADS + D model (AUC 0.941; *p* > 0.05) and the standalone VI-RADS model (AUC 0.926; *p* > 0.05). The DECT + D model demonstrated non-inferiority to VI-RADS. The difference in AUC was −0.001 (95% CI: −0.0508, 0.0487), with the lower bound exceeding the pre-specified margin of −0.10 (one-sided *p* < 0.05). Post-hoc power analysis indicated a statistical power of 98.78% for the non-inferiority hypothesis. Furthermore, VI-RADS + D yielded the numerically highest AUC, but its incremental value over standalone VI-RADS was not statistically significant (*p* > 0.05).

PR curve analysis (Fig. 4b) evaluated model performance under class imbalance. VI-RADS + D yielded the highest PR-AUC (0.918), followed by VI-RADS (0.896). Notably, DECT + D achieved 0.871, comparable to VI-RADS. In contrast, standalone DECT and radiologists performed suboptimally (PR-AUCs: 0.589, 0.573, 0.502). The substantial PR-AUC increase (0.282) in DECT + D underscores D’s pivotal value in mitigating false positives and enhancing accuracy. The calibration curves (Fig. 4c) closely adhered to the diagonal line for all prediction models and radiologist assessments, demonstrating good calibration. Furthermore, DCA quantified the clinical utility of the models (Fig. 4d). Excluding VI-RADS + D, the VI-RADS model consistently demonstrated the highest net benefit across most threshold probabilities, confirming its status as the optimal diagnostic imaging tool. Conversely, while standalone DECT yielded a limited net benefit (20%–70%), DECT + D exhibited significant clinical superiority over radiologist assessments. Notably, in the lower threshold range (0%–30%), DECT+D’s net benefit was comparable to VI-RADS. Moreover, its curve consistently exceeded the default strategies (“treat-all”/“treat-none”) across a broad range (0%–90%). These results indicate that while VI-RADS offers slightly higher benefit and simplicity, DECT + D provides a net benefit significantly superior to default strategies for MRI contraindications, establishing it as a reliable alternative.

**Fig. 5:**
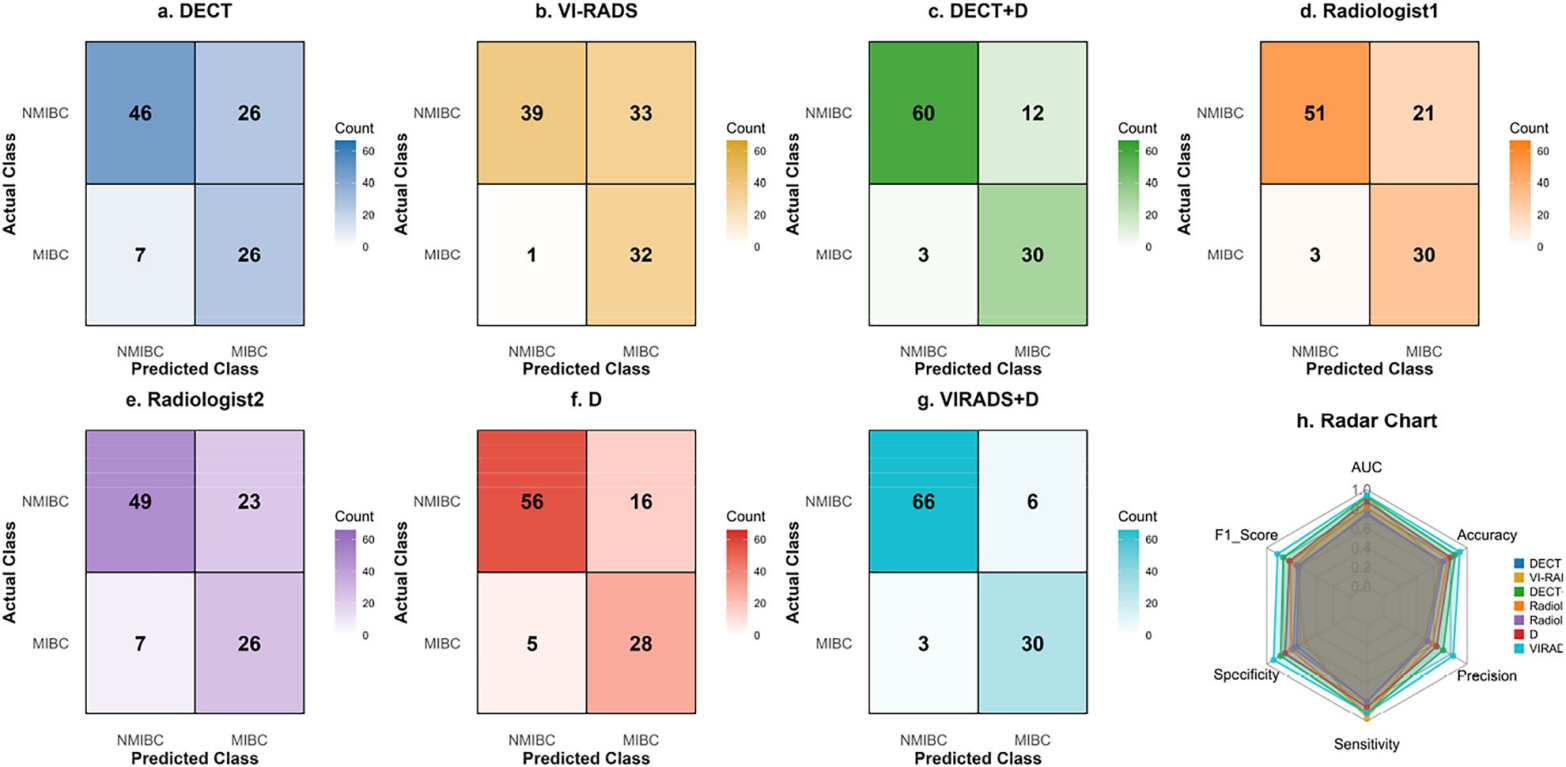
Confusion matrices (**a** DECT, **b** VI-RADS, **c** DECT + D, **d** Radiologist1, **e** Radiologist2, **f** D, **g** VIRADS + D) and radar chart (**h**) for performance comparison in assessing muscle invasion of bladder cancer

Table 3 and Fig. 5 summarize diagnostic performance variations in sensitivity and specificity across models and expert radiologists. The DECT model exhibited a sensitivity of 0.788 and a specificity of 0.639. VI-RADS (cutoff ≥ 3) yielded the highest sensitivity (0.970), but at the cost of the lowest specificity (0.542). Integration of *D* consistently enhanced performance: DECT + D achieved sensitivity 0.909 and specificity 0.833. Ultimately, VI-RADS + D attained the optimal balance (sensitivity 0.909, highest specificity 0.917). Expert evaluations showed Radiologist 1 with high sensitivity (0.909) and moderate specificity (0.708), while Radiologist 2 demonstrated sensitivity of 0.788 and specificity of 0.681.

**Table 3:** Performance comparison of different classification models and radiologists

Model	AUC (95% CI)	Accuracy	Precision	Sensitivity	Specificity	F1 score
DECT	0.761 (0.664–0.859)	0.686	0.500	0.788	0.639	0.612
DECT + D	0.925 (0.874–0.975)	0.857	0.714	0.909	0.833	0.800
Radiologist1	0.809 (0.736–0.881)	0.771	0.588	0.909	0.708	0.714
Radiologist2	0.734 (0.645–0.823)	0.714	0.531	0.788	0.681	0.634
VI-RADS	0.926 (0.871–0.980)	0.676	0.492	0.970	0.542	0.653
D	0.876 (0.804–0.948)	0.800	0.636	0.848	0.778	0.727
VI-RADS + D	0.941 (0.889–0.993)	0.914	0.833	0.909	0.917	0.870

*AUC* area under the curve, *CI* confidence interval

### VI-RADS 3–4 subgroup analysis (*n* = 44)

The analysis focused on the clinically ambiguous VI-RADS 3 and 4 subgroup. In this cohort (Fig. 4e), the VI-RADS model exhibited limited discriminatory ability (AUC: 0.652, 95% CI: 0.482–0.821). The DECT model performed notably better (AUC: 0.824, 95% CI: 0.690–0.958), although the difference in AUCs was not statistically significant (*p* > 0.05). The combined DECT + D model achieved the optimal performance among all classifiers (AUC: 0.904, 95% CI: 0.812–0.995). However, this performance was statistically comparable to the standalone DECT model (*p* > 0.05). Crucially, the DECT + D combination demonstrated significant superiority, outperforming Radiologist 1 (AUC: 0.667), Radiologist 2 (AUC: 0.545), and the single parameter *D* (AUC: 0.722) (*p* < 0.05).

The DECT + D model achieved perfect sensitivity (1.000) and specificity of 0.667, offering a reliable pathway to rule out MIBC (Table 4). Standalone DECT also presented a strong, balanced profile (sensitivity 0.818, specificity 0.727). This analysis exposed the inherent subjectivity and instability of human interpretation in this “gray zone”: The standard VI-RADS model’s efficacy was limited (sensitivity 0.636, specificity 0.667), and the two radiologists showed marked variability: Radiologist 1 achieved high sensitivity (0.818) but low specificity (0.515), while Radiologist 2’s performance was poor.

**Table 4:** Performance comparison of different classification models and radiologists in the VI-RADS 3-4 subgroup

Model	AUC (95% CI)	Accuracy	Precision	Sensitivity	Specificity	F1 score
DECT	0.824 (0.690–0.958)	0.750	0.500	0.818	0.727	0.621
DECT + D	0.904 (0.812–0.995)	0.750	0.500	1.000	0.667	0.667
Radiologist1	0.667 (0.519–0.814)	0.591	0.360	0.818	0.515	0.500
Radiologist2	0.545 (0.369–0.722)	0.455	0.217	0.455	0.455	0.294
VI-RADS	0.652 (0.482–0.821)	0.659	0.389	0.636	0.667	0.483
D	0.722 (0.532–0.911)	0.636	0.391	0.818	0.576	0.529
VI-RADS + D	0.740 (0.550–0.930)	0.773	0.529	0.818	0.758	0.643

### *D* < 3 cm subgroup analysis (*n* = 71)

Given the European Association of Urology guidelines [22] linking lesions > 3 cm to increased muscle invasion risk, a subgroup analysis was performed for the 71 lesions with *D* < 3 cm. Figure 4f shows the DECT model demonstrated robust diagnostic efficacy (AUC: 0.857, 95% CI: 0.721–0.994), statistically comparable to the VI-RADS model (AUC: 0.866, 95% CI: 0.729–1.000) (*p* > 0.05). The DECT + D combination model demonstrated the highest discriminatory ability (AUC: 0.895, 95% CI: 0.803–0.987), though the difference from the standalone DECT model was not statistically significant (*p* > 0.05). The poorest performer was Radiologist 2 (AUC: 0.652, 95% CI: 0.482–0.823).

Table 5 highlights VI-RADS limitations: high sensitivity (0.900) was compromised by low specificity (0.426), causing an excessive false-positive rate. Conversely, DECT-based models provided superior diagnostic equilibrium. Both DECT (sensitivity 0.700, specificity 0.934) and DECT + D (sensitivity 0.800, specificity 0.869) maintained high specificity, crucial for non-invasive disease identification. Quantitative augmentation was further substantiated by the VI-RADS + D model, which attained the subgroup’s highest specificity (0.984) while preserving good sensitivity (0.700).

**Table 5:** Performance comparison of different classification models and radiologists in the subgroup with lesion diameter < 3 cm

Model	AUC (95% CI)	Accuracy	Precision	Sensitivity	Specificity	F1 score
DECT	0.857 (0.721–0.994)	0.901	0.636	0.700	0.934	0.667
DECT + D	0.895 (0.803–0.987)	0.859	0.500	0.800	0.869	0.615
Radiologist1	0.761 (0.618–0.903)	0.732	0.320	0.800	0.721	0.457
Radiologist2	0.652 (0.482–0.823)	0.690	0.250	0.600	0.705	0.353
VI-RADS	0.866 (0.729–1.000)	0.493	0.205	0.900	0.426	0.333
D	0.784 (0.629–0.940)	0.789	0.368	0.700	0.803	0.483
VI-RADS + D	0.870 (0.731–1.000)	0.944	0.875	0.700	0.984	0.778

### Model interpretability and nomogram development

**Fig. 6:**
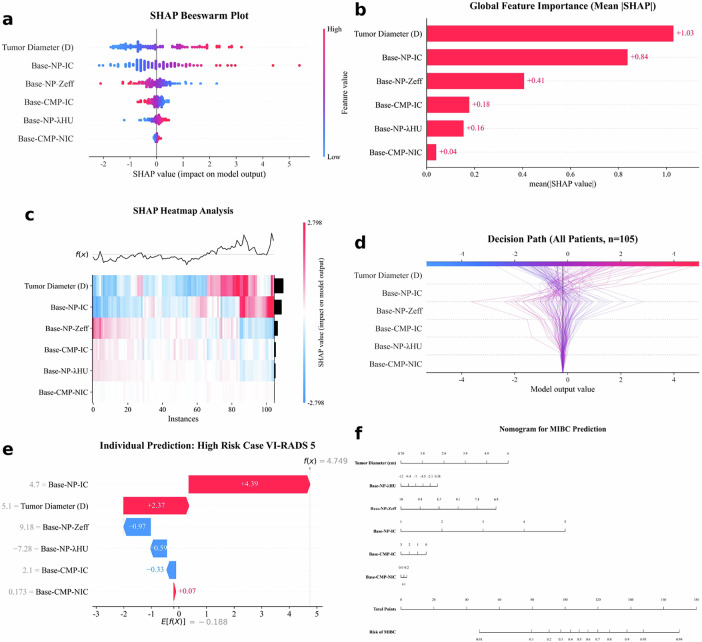
Multidimensional visualization of model interpretability and the clinical nomogram. **a** SHAP beeswarm plot. Each dot represents a patient. Red dots indicate higher feature values, corresponding to positive SHAP values (increased risk). This confirms the positive correlation between iodine concentration/tumor diameter and muscle invasion. **b** Global feature importance bar chart. Predictors are ranked by mean absolute SHAP value, identifying Tumor Diameter (D) and Base-NP-IC as the dominant features. **c** SHAP heatmap analysis. Visualizes feature attribution patterns across the population, revealing how specific combinations of DECT parameters and tumor diameter cluster to drive predictions. **d** Decision path plot. Shows cumulative decision processes for all 105 patients. The clear separation between high-risk (red trajectories) and low-risk (blue trajectories) confirms robust model discrimination. **e** Individual waterfall plot (representative VI-RADS 5 case). Details the decision logic for a high-risk patient with pathologically confirmed MIBC, where elevated Base-NP-IC (+ 4.39) and tumor diameter (+ 2.37) significantly drive the prediction. **f** DECT-based nomogram. A clinical tool integrating DECT parameters and tumor diameter for personalized MIBC risk estimation

To visualize model interpretability, SHAP analysis quantified global feature importance and individual decision paths (Fig. 6a–e). Global analysis identified D and Base-NP-IC as the predominant predictors (Fig. 6a, b), confirming that larger D and higher IC are positively associated with MIBC risk. Robust discrimination was corroborated by population-level analysis (Fig. 6c, d), showing clear stratification between high-risk and low-risk trajectories. At the individual level, feature contribution was quantified for a representative VI-RADS 5 case (Fig. 6e). Subsequently, a nomogram was developed based on the independent predictors. This nomogram (Fig. 6f) provides a graphical tool for calculating the probability of MIBC using routinely acquired DECT parameters. To further facilitate clinical application and accessibility, an interactive online web-based risk calculator was also developed. This tool is accessible at: https://huggingface.co/spaces/Mingyang3670/MIBC-Risk-Calculator.

## Discussion

This study developed a quantitative DECT model for assessing MIBC, compared against VI-RADS. While standalone DECT showed moderate discrimination (AUC 0.761), significantly inferior to VI-RADS (AUC 0.926; *p* < 0.05), the DECT + D model significantly improved performance (AUC 0.925), becoming comparable to VI-RADS (*p* > 0.05). In the equivocal VI-RADS 3–4 subgroup, DECT demonstrated robust efficacy (AUC 0.824) vs VI-RADS (AUC 0.652; *p* > 0.05). Similarly, for *D* < 3 cm, DECT (AUC 0.857) paralleled VI-RADS (AUC 0.866; *p* > 0.05). Thus, quantitative DECT offers a valuable objective complement to VI-RADS, particularly for reducing diagnostic uncertainty in equivocal cases and minimizing overstaging in small lesions.

The superior diagnostic performance of the combined DECT + D model stems from the synergistic integration of macroscopic and microscopic parameters. While D indicates aggressiveness, it offers limited insight into the tumor’s complex internal pathophysiology. Crucially, our multivariable analysis identified six independent predictors of MIBC, including D and five DECT parameters: Base-CMP-IC, Base-CMP-NIC, Base-NP-λ_HU_, Base-NP-Z_eff_, and Base-NP-IC. The specific localization of these key predictors provides compelling evidence that the tumor base region harbors critical imaging information for predicting invasion. These findings are consistent with Zheng et al [23], who demonstrated the importance of radiomic features from this specific region for accurate assessment of muscle invasion. Mechanistically, these quantitative parameters serve as surrogates for specific biological processes: IC and NIC quantify the profuse neoangiogenesis supporting invasive growth, while λ_HU_ and Z_eff_ capture alterations in material composition and tissue heterogeneity caused by tumor infiltration into the muscularis propria. Thus, combining D with quantitative base characterization enables comprehensive MIBC assessment.

Our findings regarding the diagnostic performance of VI-RADS (AUC 0.926) demonstrate remarkable consistency with a recent diagnostic meta-analysis by Luo et al [24] (pooled AUC 0.93). This concordance reaffirms multiparametric MRI as the reference standard and validates our study’s baseline interpretation reliability. However, while VI-RADS in our cohort showed superior sensitivity (0.970 vs 0.90), its specificity was low (0.542 vs 0.86). This discrepancy highlights qualitative MRI’s limitation: inherent subjectivity may lead to cautious interpretation, increasing false positives and overstaging risk. The combined DECT + D model demonstrated an optimized sensitivity-specificity trade-off. It achieved an AUC (0.925) comparable to the meta-analysis pooled estimate, delivering a robust profile. Its sensitivity (0.909) aligns perfectly with the meta-analysis pooled sensitivity (0.90), reliably detecting muscle invasion. Critically, this was achieved with a significantly improved specificity of 0.833. This suggests that integrating tumor size with objective quantitative parameters mitigates the overestimation tendency observed in visual scoring. Compared to historical conventional CT studies, which reported relatively low staging accuracy (35–55%) [25], our multiparametric DECT approach demonstrates a substantial improvement, suggesting its potential as a reliable and objective tool for preoperative staging.

Hu et al [26] reported that their DECT model’s AUC was 0.704, with only NIC identified as an independent predictor. In contrast, our multiparametric integration strategy yielded a standalone DECT AUC of 0.761, which increased to 0.925 when combined with D. This discrepancy likely stems from our innovative design—focusing parameter extraction on the tumor base, rather than Hu et al’s “whole-tumor volume” segmentation. Notably, even Hu et al's complex fusion model (incorporating high-dimensional texture features) achieved a slightly lower test AUC (0.893) than our DECT + D model (0.925). Our integration of tumor base-focused interpretable DECT parameters, and *D*, more directly and accurately quantifies tumor invasive behavior than radiomics methods relying on whole-tumor texture. Furthermore, in the *D* < 3 cm subgroup, our standalone DECT (AUC 0.857) and combined DECT + D (AUC 0.895) models both outperformed Hu et al's single-parameter DECT model (AUC 0.726) in the equivalent test cohort.

This study investigated the diagnostic efficacy within the VI-RADS 3–4 “gray zone,” where MIBC risk uncertainty poses a critical challenge. The necessity for resolving this uncertainty is strongly corroborated by a prospective multicenter study: findings showed that MIBC risk reached 45.8% and 69.6% for VI-RADS 3 and 4 patients, respectively, following initial TURBT [27]. Following a second TURBT, these critical risk ratios escalated further to 58.3% and 87.0% [27]. Wang et al [28] proposed a VI-RADS_TCL strategy, hybridizing Tumor Contact Length (TCL) with the VI-RADS score to resolve the diagnostic difficulty of VI-RADS 3 lesions. This successfully reduced the false-positive rate while retaining sensitivity, achieving significantly greater specificity (82.46%–87.72%) and positive predictive values (90.91%–91.59%) than the standard VI-RADS score (*p* < 0.05). Similarly, Yu et al [29] developed an MRI nomogram integrating three key features (TCL > max diameter, flat morphology, and lower standard deviation of apparent diffusion coefficient) to predict MIBC in VI-RADS 3 patients. In the validation cohort, this nomogram demonstrated an AUC of 0.885, accuracy of 0.817, sensitivity of 0.900, and specificity of 0.784. Nevertheless, despite the promise of these studies, the VI-RADS assessment remains suboptimal in the VI-RADS 3–4 subgroup (AUC = 0.652). This qualitative limitation due to subjective morphological features impedes precise risk stratification. The robust quantitative models developed in our study offer a superior solution: the combined DECT + D model achieved the strongest discriminatory ability (AUC = 0.904), outperforming Yu et al’s nomogram. These results confirm the power of the DECT-based model in providing a reliable, quantifiable solution for the VI-RADS “gray zone” diagnostic dilemma.

Despite promising findings, several inherent limitations must be acknowledged. First, all data were collected from a single tertiary referral center, restricting external generalizability. This setting introduced spectrum bias with a high proportion of VI-RADS 5 cases. These advanced lesions skewed the diagnostic spectrum, creating a non-generalizable VI-RADS 3–4 subgroup and difficulties in qualitative radiological assessment. Performance in this small subgroup may not reflect routine clinical practice. This subgroup analysis is thus exploratory and hypothesis-generating for future prospective studies. Second, to address potential subjectivity in manual ROI delineation, we used the average values from two independent radiologists to minimize inter-observer variability. Third, despite multiple tumor-based samplings, TURBT carries an inherent risk of understaging due to limited sampling depth. Finally, our study lacks external validation. Prospective, multicenter validation is essential to confirm the DECT-based model’s robustness and generalizability across diverse populations and imaging platforms.

Although VI-RADS remains the current diagnostic standard, the DECT-based model developed in this study, integrating tumor diameter and DECT parameters, achieved performance statistically comparable to the established VI-RADS standard. Crucially, the model demonstrated superior stability in clinically challenging scenarios: it provided essential objective quantification for the equivocal VI-RADS 3–4 subgroup and offered a safeguard against overstaging for small lesions. Supported by the developed nomogram and web-based risk calculator, this DECT-based approach serves as a highly accurate and viable alternative for preoperative staging, particularly for patients with MRI contraindications.

## Supplementary information

ELECTRONIC SUPPLEMENTARY MATERIAL

## Data Availability

Due to ethical restrictions on patient privacy, the raw data are not publicly available. However, de-identified datasets may be obtained from the corresponding author upon reasonable request, subject to approval by the institutional ethics committee.

## Abbreviations

AUC: Area under the curve
CI: Confidence interval
CTU: CT urography
DCA: Decision curve analysis
D: Diameter
DWI: Diffusion-weighted imaging
DECT: Dual-energy CT
DCE: Dynamic contrast-enhanced
GSI: Gemstone spectral imaging
ICC: Intraclass correlation coefficient
MIBC: Muscle-invasive bladder cancer
NMIBC: Non-muscle-invasive bladder cancer
PR: Precision-recall
ROC: Receiver operating characteristic
ROI: Region of interest
T_2_WI: T_2_-weighted imaging
TURBT: Transurethral resection of bladder tumor
VI-RADS: Vesical imaging reporting and data system

## References

[1] Sung H, Ferlay J, Siegel RL et al (2021) Global Cancer Statistics 2020: GLOBOCAN estimates of incidence and mortality worldwide for 36 cancers in 185 countries. CA Cancer J Clin 71:209–249. 10.3322/caac.21660 33538338 10.3322/caac.21660

[2] Witjes JA, Bruins HM, Cathomas R et al (2021) European Association of Urology Guidelines on muscle-invasive and metastatic bladder cancer: summary of the 2020 guidelines. Eur Urol 79:82–104. 10.1016/j.eururo.2020.03.055 32360052 10.1016/j.eururo.2020.03.055

[3] Netto GJ, Amin MB, Berney DM et al (2022) The 2022 World Health Organization Classification of tumors of the urinary system and male genital organs-part B: prostate and urinary tract tumors. Eur Urol 82:469–482. 10.1016/j.eururo.2022.07.002 35965208 10.1016/j.eururo.2022.07.002

[4] Kamat AM, Hahn NM, Efstathiou JA et al (2016) Bladder cancer. Lancet 388:2796–2810. 10.1016/S0140-6736(16)30512-8 27345655 10.1016/S0140-6736(16)30512-8

[5] Alfred Witjes J, Max Bruins H, Carrión A et al (2024) European Association of Urology Guidelines on muscle-invasive and metastatic bladder cancer: summary of the 2023 guidelines. Eur Urol 85:17–31. 10.1016/j.eururo.2023.08.016 37858453 10.1016/j.eururo.2023.08.016

[6] Babjuk M, Böhle A, Burger M et al (2017) EAU guidelines on non-muscle-invasive urothelial carcinoma of the bladder: update 2016. Eur Urol 71:447–461. 10.1016/j.eururo.2016.05.041 27324428 10.1016/j.eururo.2016.05.041

[7] Herr H (2019) Preventable cancer deaths associated with bladder preservation for muscle invasive bladder cancer. Urology 130:20–21. 10.1016/j.urology.2019.04.032 31071350 10.1016/j.urology.2019.04.032

[8] Mally D, Paffenholz P (2020) [Complication management for TUR of the bladder]. Aktuelle Urol 51:450–455. 10.1055/a-1210-2163 32785917 10.1055/a-1210-2163

[9] Gontero P, Sylvester R, Pisano F et al (2015) Prognostic factors and risk groups in T1G3 non-muscle-invasive bladder cancer patients initially treated with Bacillus Calmette-Guérin: results of a retrospective multicenter study of 2451 patients. Eur Urol 67:74–82. 10.1016/j.eururo.2014.06.040 25043942 10.1016/j.eururo.2014.06.040

[10] Mariappan P, Zachou A, Grigor KM, Edinburgh Uro-Oncology Group (2010) Detrusor muscle in the first, apparently complete transurethral resection of bladder tumour specimen is a surrogate marker of resection quality, predicts risk of early recurrence, and is dependent on operator experience. Eur Urol 57:843–849. 10.1016/j.eururo.2009.05.047 19524354 10.1016/j.eururo.2009.05.047

[11] Cumberbatch MGK, Foerster B, Catto JWF et al (2018) Repeat transurethral resection in non-muscle-invasive bladder cancer: a systematic review. Eur Urol 73:925–933. 10.1016/j.eururo.2018.02.014 29523366 10.1016/j.eururo.2018.02.014

[12] Panebianco V, Narumi Y, Altun E et al (2018) Multiparametric magnetic resonance imaging for bladder cancer: development of VI-RADS (vesical imaging-reporting and data system). Eur Urol 74:294–306. 10.1016/j.eururo.2018.04.029 29755006 10.1016/j.eururo.2018.04.029 PMC6690492

[13] Marchioni M, Primiceri G, Delli Pizzi A et al (2020) Could bladder multiparametric MRI be introduced in routine clinical practice? Role of the new VI-RADS score: results from a prospective study. Clin Genitourin Cancer 18:409–415.e1. 10.1016/j.clgc.2020.03.002 32273236 10.1016/j.clgc.2020.03.002

[14] Barchetti G, Simone G, Ceravolo I et al (2019) Multiparametric MRI of the bladder: inter-observer agreement and accuracy with the vesical imaging-reporting and data system (VI-RADS) at a single reference center. Eur Radiol 29:5498–5506. 10.1007/s00330-019-06117-8 30887202 10.1007/s00330-019-06117-8

[15] Ueno Y, Takeuchi M, Tamada T et al (2019) Diagnostic accuracy and interobserver agreement for the vesical imaging-reporting and data system for muscle-invasive bladder cancer: a multireader validation study. Eur Urol 76:54–56. 10.1016/j.eururo.2019.03.012 30922688 10.1016/j.eururo.2019.03.012

[16] Johnson TRC (2012) Dual-energy CT: general principles. AJR Am J Roentgenol 199:S3–S8. 10.2214/AJR.12.9116 23097165 10.2214/AJR.12.9116

[17] Siegel MJ, Kaza RK, Bolus DN et al (2016) White paper of the society of computed body tomography and magnetic resonance on dual-energy CT, part 1: technology and terminology. J Comput Assist Tomogr 40:841–845. 10.1097/RCT.0000000000000531 27841774 10.1097/RCT.0000000000000531

[18] Hamid S, Nasir MU, So A, Andrews G, Nicolaou S, Qamar SR (2021) Clinical applications of dual-energy CT. Korean J Radiol 22:970–982. 10.3348/kjr.2020.0996 33856133 10.3348/kjr.2020.0996 PMC8154785

[19] Li J, Fang M, Wang R et al (2018) Diagnostic accuracy of dual-energy CT-based nomograms to predict lymph node metastasis in gastric cancer. Eur Radiol 28:5241–5249. 10.1007/s00330-018-5483-2 29869176 10.1007/s00330-018-5483-2

[20] Lewin M, Laurent-Bellue A, Desterke C et al (2022) Evaluation of perfusion CT and dual-energy CT for predicting microvascular invasion of hepatocellular carcinoma. Abdom Radiol (NY) 47:2115–2127. 10.1007/s00261-022-03511-7 35419748 10.1007/s00261-022-03511-7

[21] Zhan Y, Wang Y, Wang P et al (2023) Pretreatment dual-energy CT for predicting early response to induction chemotherapy and survival in nasopharyngeal carcinoma. Eur Radiol 33:9052–9062. 10.1007/s00330-023-09837-0 37405505 10.1007/s00330-023-09837-0

[22] Babjuk M, Burger M, Capoun O et al (2022) European Association of Urology Guidelines on non-muscle-invasive Bladder Cancer (Ta, T1, and Carcinoma in situ). Eur Urol 81:75–94. 10.1016/j.eururo.2021.08.010 34511303 10.1016/j.eururo.2021.08.010

[23] Zheng J, Kong J, Wu S et al (2019) Development of a noninvasive tool to preoperatively evaluate the muscular invasiveness of bladder cancer using a radiomics approach. Cancer 125:4388–4398. 10.1002/cncr.32490 31469418 10.1002/cncr.32490

[24] Luo C, Huang B, Wu Y, Chen J, Chen L (2020) Use of vesical imaging-reporting and data system (VI-RADS) for detecting the muscle invasion of bladder cancer: a diagnostic meta-analysis. Eur Radiol 30:4606–4614. 10.1007/s00330-020-06802-z 32242273 10.1007/s00330-020-06802-z

[25] Bostrom PJ, Rhijn BWG, van, Fleshner N et al (2010) Staging and staging errors in bladder cancer. Eur Urol Suppl 9:2–9. 10.1016/j.eursup.2010.01.005

[26] Hu M, Wei W, Zhang J et al (2024) Assessing muscle invasion in bladder cancer via virtual biopsy: a study on quantitative parameters and classical radiomics features from dual-energy CT imaging. BMC Med Imaging 24: 245. 10.1186/s12880-024-01427-w 39285354 10.1186/s12880-024-01427-w PMC11403826

[27] Metwally MI, Zeed NA, Hamed EM et al (2021) The validity, reliability, and reviewer acceptance of VI-RADS in assessing muscle invasion by bladder cancer: a multicenter prospective study. Eur Radiol 31:6949–6961. 10.1007/s00330-021-07765-5 33606105 10.1007/s00330-021-07765-5

[28] Wang X, Tu N, Sun F et al (2021) Detecting muscle invasion of bladder cancer using a proposed magnetic resonance imaging strategy. J Magn Reson Imaging 54:1212–1221. 10.1002/jmri.27676 33998725 10.1002/jmri.27676

[29] Yu R, Cai L, Cao Q et al (2024) Development and validation of an MRI-based nomogram for preoperative detection of muscle invasion in VI-RADS 3. J Magn Reson Imaging: JMRI 60:448–457. 10.1002/jmri.29103 37902432 10.1002/jmri.29103

