# Supplementary material for: Assessment of multi-parameter dual-energy CT in predicting muscle invasion in bladder cancer: comparison with VI-RADS
Source: Insights Imaging. 2026 Apr 8;17:96. doi: 10.1186/s13244-026-02275-8 (PMC13062054; doi:10.1186/s13244-026-02275-8)
Supplement: Supplementary file 1 — ELECTRONIC SUPPLEMENTARY MATERIAL [file 13244_2026_2275_MOESM1_ESM.pdf]

Assessment of Multi-Parameter Dual-Energy CT in Predicting Muscle Invasion in Bladder Cancer: Comparison with VI-RADS

ELECTRONIC SUPPLEMENTARY MATERIAL

Table S1 MRI Scan Parameters

	TR (ms)	TE (ms)	Flip angle (°)	FOV (cm)	Slice thickness (mm)	Slice gap (mm)	b values (s/mm²)
**Sag T_2WI**	7562	85	125	22	3	1	–
**Cor T_2WI**	8049	85	125	24	3	1	–
**Ax T_2WI**	8049	85	125	24	3	1	–
**Sag DWI**	2937	–	–	28	3.6	1	0, 800
**Ax DWI**	2742	–	–	28	3.6	1	0, 800
**Ax T_1WI**	7.6	2.3	12	24	3	–	–
**Sag DCE**	9.1	2.5	12	24	3	–	–

Note: T2-weighted imaging (T_2WI); T1-weighted imaging (T_1WI); Diffusion-weighted imaging (DWI); Dynamic contrast-enhanced (DCE)

Table S2 List of abbreviations and full names for dual-energy CT quantitative parameters.

Abbreviation	Full Name
Tumor-CMP-CT_{40keV}	40 keV CT value of tumor in corticomedullary phase
Tumor-CMP-CT_{70keV}	70 keV CT value of tumor in corticomedullary phase
Tumor-CMP-λ_{HU}	Spectral HU curve slope of tumor in corticomedullary phase
Tumor-CMP-Z_{eff}	Effective atomic number of tumor in corticomedullary phase
Tumor-CMP-IC	Iodine concentration of tumor in corticomedullary phase
Tumor-CMP-NIC	Normalized iodine concentration of tumor in corticomedullary phase
Base-CMP-CT_{40keV}	40 keV CT value of tumor base in corticomedullary phase

Abbreviation	Full Name
Base-CMP-CT_{70keV}	70 keV CT value of tumor base in corticomedullary phase
Base-CMP-λ_{HU}	Spectral HU curve slope of tumor base in corticomedullary phase
Base-CMP-Z_{eff}	Effective atomic number of tumor base in corticomedullary phase
Base-CMP-IC	Iodine concentration of tumor base in corticomedullary phase
Base-CMP-NIC	Normalized iodine concentration of tumor base in corticomedullary phase
Tumor-NP-CT_{40keV}	40 keV CT value of tumor in nephrographic phase
Tumor-NP-CT_{70keV}	70 keV CT value of tumor in nephrographic phase
Tumor-NP-λ_{HU}	Spectral HU curve slope of tumor in nephrographic phase
Tumor-NP-Z_{eff}	Effective atomic number of tumor in nephrographic phase
Tumor-NP-IC	Iodine concentration of tumor in nephrographic phase
Tumor-NP-NIC	Normalized iodine concentration of tumor in nephrographic phase
Base-NP-CT_{40keV}	40 keV CT value of tumor base in nephrographic phase
Base-NP-CT_{70keV}	70 keV CT value of tumor base in nephrographic phase
Base-NP-λ_{HU}	Spectral HU curve slope of tumor base in nephrographic phase
Base-NP-Z_{eff}	Effective atomic number of tumor base in nephrographic phase
Base-NP-IC	Iodine concentration of tumor base in nephrographic phase
Base-NP-NIC	Normalized iodine concentration of tumor base in nephrographic phase

Note: The nomenclature follows the format [Region]-[Phase]-[Metric]

Table S3 Inter-observer reliability of DECT parameters: ICC with 95% CIs

DECT Parameters	ICC (95%CI)
Tumor-CMP-CT_{40keV} (HU)	0.706 (0.582–0.827)
Tumor-CMP-CT_{70keV} (HU)	0.725 (0.618–0.826)
Tumor-CMP-λ_{HU}	0.692 (0.567–0.806)
Tumor-CMP-Z_{eff}	0.680 (0.552–0.778)

DECT Parameters	ICC (95%CI)
Tumor-CMP-IC (mg/ml)	0.724 (0.616–0.812)
Tumor-CMP-NIC (%)	0.706 (0.598–0.825)
Base-CMP-CT_{40keV} (HU)	0.691 (0.582–0.793)
Base-CMP-CT_{70keV} (HU)	0.685 (0.580–0.789)
Base-CMP-λ_{HU}	0.718 (0.596–0.819)
Base-CMP-Z_{eff}	0.688 (0.592–0.778)
Base-CMP-IC (mg/ml)	0.741 (0.600–0.837)
Base-CMP-NIC (%)	0.755 (0.628–0.837)
Tumor-NP-CT_{40keV} (HU)	0.692 (0.609–0.772)
Tumor-NP-CT_{70keV} (HU)	0.747 (0.617–0.833)
Tumor-NP-λ_{HU}	0.724 (0.607–0.862)
Tumor-NP-Z_{eff}	0.732 (0.596–0.818)
Tumor-NP-IC (mg/ml)	0.713 (0.605–0.821)
Tumor-NP-NIC (%)	0.694 (0.596–0.792)
Base-NP-CT_{40keV} (HU)	0.712 (0.608–0.815)
Base-NP-CT_{70keV} (HU)	0.682 (0.598–0.804)
Base-NP-λ_{HU}	0.692 (0.574–0.800)
Base-NP-Z_{eff}	0.677 (0.563–0.786)
Base-NP-IC (mg/ml)	0.704 (0.587–0.806)
Base-NP-NIC (%)	0.691 (0.588–0.819)

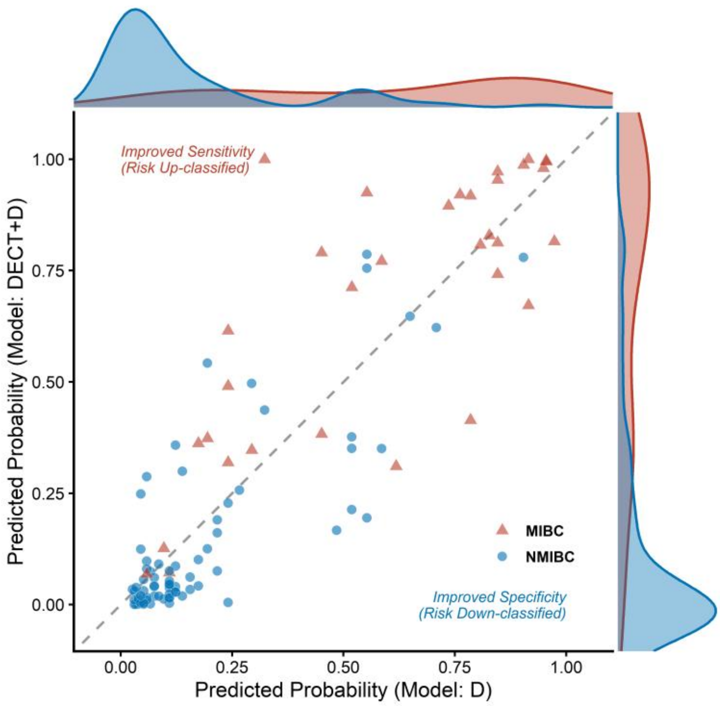

Fig. S1 Reclassification scatter plot of predicted probabilities from the D model versus the DECT+D model. The x-axis shows the predicted probability of MIBC using D alone, and the y-axis shows the probability using the combined DECT+D model. Red triangles represent patients with MIBC; blue circles represent patients with NMIBC. Points above the dashed diagonal line indicate up-classification and points below indicate down-classification by the DECT+D model.

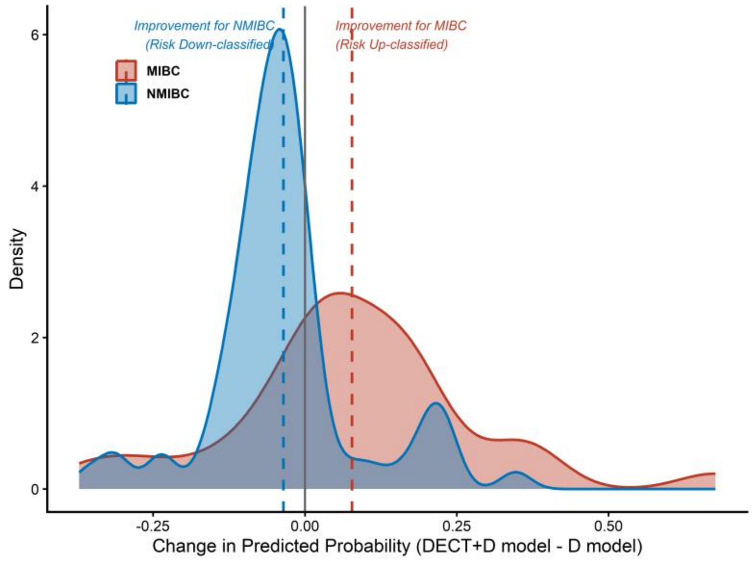

Fig. S2 Distribution of changes in predicted probabilities of MIBC predicted by

the DECT+D model compared with the D model alone (integrated discrimination improvement [IDI] analysis).Kernel density plots show the difference in predicted probability (DECT+D model minus D model). Positive values indicate higher predicted risk with the DECT+D model. The blue curve represents patients with NMIBC and the red curve represents patients with MIBC. Dashed vertical lines indicate the mean change in predicted probability for each group.

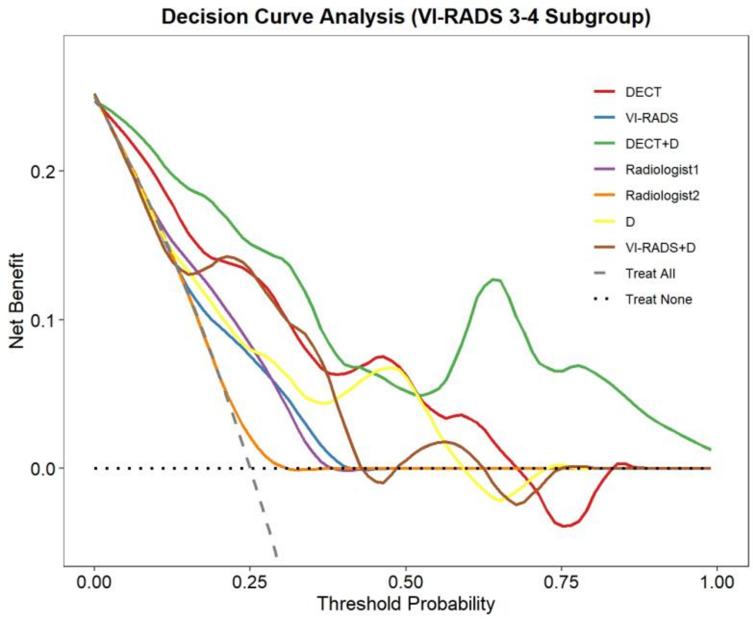

Fig. S3 Decision curve analysis in the subgroup with VI-RADS 3–4.

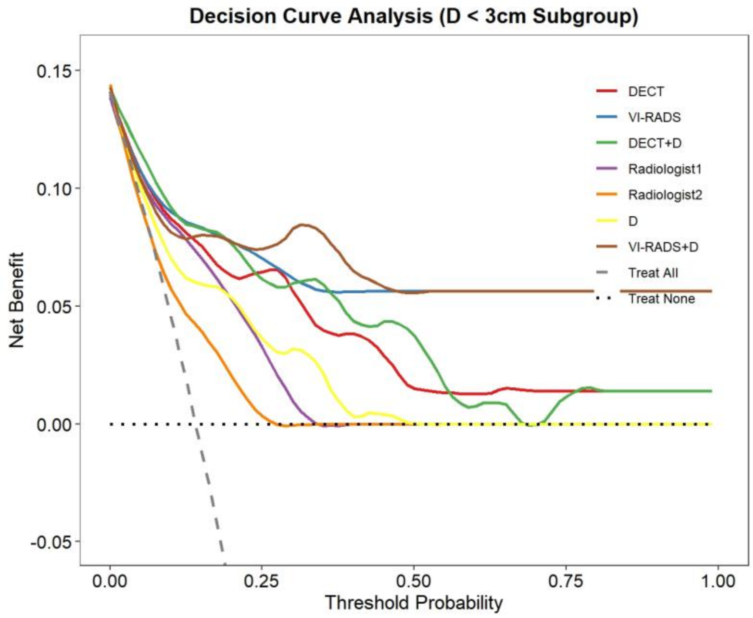

Fig. S4 Decision curve analysis in the subgroup with D < 3cm.
